# Supplementary material for: HIVprotI: an integrated web based platform for prediction and design of HIV proteins inhibitors
Source: J Cheminform. 2018 Mar 9;10:12. doi: 10.1186/s13321-018-0266-y (PMC5845081; doi:10.1186/s13321-018-0266-y)
Supplement: Supplementary file 2 — Additional file 2. Source code of HIVProtI web server. [file 13321_2018_266_MOESM2_ESM.zip › HIVprotI_Source-code/hivprotI/weka-3-6-6/doc/weka/core/RevisionUtils.html]

RevisionUtils


---


|  |  |  |
| --- | --- | --- |
| **Overview** \| **Package** \| **Class** \| **Tree** \| **Deprecated** \| **Index** \| **Help** \| **Weka's home** | |  |
| **PREV CLASS**   **NEXT CLASS** | **FRAMES**    **NO FRAMES**     **All Classes** |  |
| SUMMARY: NESTED \| FIELD \| CONSTR \| METHOD | DETAIL: FIELD \| CONSTR \| METHOD |  |


---


## weka.core Class RevisionUtils

```
java.lang.Object
  weka.core.RevisionUtils
```

---

``` public class RevisionUtils extends java.lang.Object ```

Contains utility functions for handling revisions.

**Version:**
:   $Revision: 1.1 $

**Author:**
:   fracpete (fracpete at waikato dot ac dot nz)

---

| **Nested Class Summary** | |
| --- | --- |
| `static class` | `RevisionUtils.Type`             Enumeration of source control types. |


**Constructor Summary**

`RevisionUtils()`


| **Method Summary** | |
| --- | --- |
| `static java.lang.String` | `extract(RevisionHandler handler)`             Extracts the revision string returned by the RevisionHandler. |
| `static java.lang.String` | `extract(java.lang.String s)`             Extracts the revision string. |
| `static RevisionUtils.Type` | `getType(RevisionHandler handler)`             Determines the type of a (sanitized) revision string returned by the RevisionHandler. |
| `static RevisionUtils.Type` | `getType(java.lang.String revision)`             Determines the type of a (sanitized) revision string. |
| `static void` | `main(java.lang.String[] args)`             For testing only. |

**Methods inherited from class java.lang.Object**

`equals, getClass, hashCode, notify, notifyAll, toString, wait, wait, wait`

**Constructor Detail**

### RevisionUtils

```
public RevisionUtils()
```


**Method Detail**

### extract

```
public static java.lang.String extract(RevisionHandler handler)
```

:   Extracts the revision string returned by the RevisionHandler.

    :   **Parameters:**: `handler` - the RevisionHandler to get the revision for **Returns:**: the actual revision string

---


### extract

```
public static java.lang.String extract(java.lang.String s)
```

:   Extracts the revision string.

    :   **Parameters:**: `s` - the string to get the revision string from **Returns:**: the actual revision string

---


### getType

```
public static RevisionUtils.Type getType(RevisionHandler handler)
```

:   Determines the type of a (sanitized) revision string returned by the
    RevisionHandler.

    :   **Parameters:**: `handler` - the RevisionHandler to determine the type for **Returns:**: the type, UNKNOWN if it cannot be determined

---


### getType

```
public static RevisionUtils.Type getType(java.lang.String revision)
```

:   Determines the type of a (sanitized) revision string. Use extract(String)
    method to extract the revision first before calling this method.

    :   **Parameters:**: `revision` - the revision to get the type for **Returns:**: the type, UNKNOWN if it cannot be determined **See Also:**: `extract(String)`

---


### main

```
public static void main(java.lang.String[] args)
                 throws java.lang.Exception
```

:   For testing only. The first parameter must be a classname of a
    class implementing the weka.core.RevisionHandler interface.

    :   **Parameters:**: `args` - the commandline arguments **Throws:**: `java.lang.Exception` - if something goes wrong


---


|  |  |  |
| --- | --- | --- |
| **Overview** \| **Package** \| **Class** \| **Tree** \| **Deprecated** \| **Index** \| **Help** \| **Weka's home** | |  |
| **PREV CLASS**   **NEXT CLASS** | **FRAMES**    **NO FRAMES**     **All Classes** |  |
| SUMMARY: NESTED \| FIELD \| CONSTR \| METHOD | DETAIL: FIELD \| CONSTR \| METHOD |  |


---
